# Fundamental Elements in Autism: From Neurogenesis and Neurite Growth to Synaptic Plasticity

**DOI:** 10.3389/fncel.2017.00359

**Published:** 2017-11-20

**Authors:** James Gilbert, Heng-Ye Man

**Affiliations:** ^1^Department of Biology, Boston University, Boston, MA, United States; ^2^Department of Pharmacology & Experimental Therapeutics, Boston University School of Medicine, Boston, MA, United States

**Keywords:** autism, ASD, developmental neurobiological disorders, neurogenesis, dendrite growth, neuron morphogenesis, synapse, synaptic plasticity

## Abstract

Autism spectrum disorder (ASD) is a set of neurodevelopmental disorders with a high prevalence and impact on society. ASDs are characterized by deficits in both social behavior and cognitive function. There is a strong genetic basis underlying ASDs that is highly heterogeneous; however, multiple studies have highlighted the involvement of key processes, including neurogenesis, neurite growth, synaptogenesis and synaptic plasticity in the pathophysiology of neurodevelopmental disorders. In this review article, we focus on the major genes and signaling pathways implicated in ASD and discuss the cellular, molecular and functional studies that have shed light on common dysregulated pathways using *in vitro*, *in vivo* and human evidence.

**Highlights**

- Autism spectrum disorder (ASD) has a prevalence of 1 in 68 children in the United States.
- ASDs are highly heterogeneous in their genetic basis.
- ASDs share common features at the cellular and molecular levels in the brain.
- Most ASD genes are implicated in neurogenesis, structural maturation, synaptogenesis and function.

## Introduction

Autism spectrum disorder (ASD) comprises a heterogeneous class of neurodevelopmental disorders characterized by impaired social interactions, restrictive interests and repetitive behaviors (Landa, 2008). ASD typically presents with other mental and physical disabilities such as anxiety, attention-deficit/hyperactivity disorder (ADHD), intellectual disability (ID), epilepsy and impairments in motor coordination. In up to 25% of individuals diagnosed with ASD, an identifiable or genetic variant can be identified, providing valuable insights into the mechanisms involved in proper neurodevelopment (Huguet et al., 2013). A large number of ASD-linked genes are also associated with broad processes such as metabolism, chromatin remodeling, mRNA regulation, protein synthesis and synaptic function.

The human brain contains about 86 billion neurons making trillions of connections (Azevedo et al., 2009). Most neurons are produced in the ventricular zone (VZ) and migrate radially out into the developing neocortex, and it is estimated that about 75% of rodent, and up to 90% of human neurons use glial-guided migration (Letinic et al., 2002). The movement of neurons along migratory routes is guided by a number of guidance molecules that direct their movement into the cortex and the formation of an organized 6-layered structure (Marín and Rubenstein, 2001; Huang, 2009; Valiente and Marin, 2010).

After neuronal migration, neurons must undergo extensive morphological changes. Long axonal processes are extended and are required to connect to target neurons with precision, while complex dendritic arbors must grow and branch to occupy specified dendritic field volumes. These processes take place during prenatal and early postnatal periods and lay the foundations for neuronal connectivity within and across brain regions. Ultimately, with activity-dependent structural remodeling, neurons form synaptic connections and incorporate into functional neuronal networks for proper brain function. It’s of no surprise that disruptions in any of these intricate processes would cause abnormalities in brain development and function, leading to neurodevelopmental disorders including ASD.

While ASD shares characteristic features at the behavioral level, its underlying causes are highly heterogeneous. Developmental dysregulation in ASD may affect processes ranging from progenitor cell proliferation and neuronal differentiation to neuron migration, axon guidance, dendrite outgrowth, synaptogenesis, synaptic function and neural circuitry.

Studies of ASD-related brain pathologies indicate that abnormal acceleration of brain growth in early childhood (Wegiel et al., 2010) accompanied with impaired neuron morphological development and brain cytoarchitecture are common features in ASDs (Bauman and Kemper, 1985; Bailey et al., 1998; van Kooten et al., 2008). Additionally, impairments in synapse formation and synaptic plasticity (Bourgeron, 2007, 2009), which ultimately lead to functional and cognitive impairments, are fundamental causative factors underlying ASD pathology.

Analysis of the Simmons Foundation Autism Research Initiative (SFARI) gene database shows that ASD causative genes display vast diversity involving up to one thousand genes (Banerjee-Basu and Packer, 2010; Abrahams et al., 2013). Additionally, a large number of rare genetic variants in protein-coding genes are causative for ASD, none of which individually account for more than 1% of the total number of ASD diagnoses. Therefore, the complexity and heterogeneity of autism genetics is a major challenge when investigating the underlying neurobiological pathways that are shared within ASD (Happe et al., 2006).

The ability to classify ASD patients according to genetics has been enhanced by the substantial amount of work done to understand ASD-linked genes, and the role of their encoded genes, in the underlying neuropathologies. For example, well known ASD-linked genes, such as neurexin, neuroligin and Shank have been well characterized for their roles in synaptic formation and function (Toro et al., 2010). Additionally, co-expression network analyses of ASD-linked genes have identified that early developmental periods when neurogenesis and synaptogenesis occur are commonly disrupted processes in ASD (Parikshak et al., 2013). Genes involved with regulating developmental processes such as Chromodomain Helicase DNA-binding protein 8 (*CHD8*), T-Box Brain Protein 1 (*TBR1*), and Fragile X Mental Retardation 1 (*FMR1*), have also been linked to ASD (Parikshak et al., 2013; Willsey et al., 2013). These findings suggest that multiple processes during prenatal and early postnatal brain growth are linked to ASD, underscoring the vast heterogeneity of causative factors leading to ASD pathogenesis. ASD-risk genes, their associated disorders and the neurodevelopmental processes they affect are shown in Table 1.

**Table 1:** Autism spectrum disorder (ASD) genes and associated disorders.

Protein	Gene symbol	SFARI score	Associated disorders	Affected developmental processes
Astrotactin 2	*ASTN2*	3	ASD, ADHD, DD, EP, ID, OCD, SCZ	Neuron migration, Cell-Cell adhesion
Autism susceptibility candidate 2	*AUTS2*	S	ASD, ADHD, DD, EP, ID, SCZ	Neuron migration, Neurite growth
Cadherin 10	*CDH10*	4	ASD	Cell-Cell adhesion
Cadherin 13	*CDH13*	N/A	ASD	Cell-Cell adhesion
Cadherin 9	*CDH9*	4	ASD	Cell-Cell adhesion
Chromodomain helicase DNA-binding protein 8	*CHD8*	1S	ASD, DD, ID, SCZ	Cell-Cell adhesion
Contactin-associated protein-like 2	*CNTNAP2*	2S	ASD, ADHD, DD, EP, ID, OCD, SCZ, TS	Neuron migration, Synapse formation, Synaptic function
Postsynaptc density 95 KDa	*DLG4*	N/A	ASD, EP, ID, SCZ	Synaptogenesis
Distal-less homeobox 1/2	*DLX1/2*	5	ASD	Neuron migration
Fragile X mental retardation gene 1	*FMR1*	S	ASD, ADHD, DD, EP, ID	Translation, Synapse Formation, Synaptic plasticity
Gephyrin	*GPHN*	3	ASD, EP, ID, SCZ	Synaptogenesis
KIDLIA	*KIAA2022/NEXMIF*	3	ASD, ID	Neurite growth
Lissencephaly 1	*LIS1*	N/A	ASD, DD, ID, EP	Neurogenesis, Neuron migration, Intracellular transport
Methyl CpG binding protein 2	*MECP2*	S	ASD, ADHD, DD, EP, ID SCZ	Transcription, Neurite growth, Synaptogenesis, Synaptic plasticity
NudE nuclear distribution E homolog 1	*NDE1*	N/A	ASD, ADHD, DD, SCZ	Neurogenesis, Neuron migration, Intracellular transport
Neurofibromatosis 1	NF1	4S	ASD	Neurite growth
Neuroligin 1/2/3/4/4Y	*NRLG1/2/3/4/4Y*	N/A		Cell-Cell adhesion, Synaptogenesis
Neurexin 1/2/3	*NRXN1/2/3*	2	ASD, ADHD, BPD, DD, EP, ID, SCZ, TS	Cell-Cell adhesion, Synaptogenesis
Phosphatase and tensin homolog	*PTEN*	1S	ASD, ADHD, DD, EP, ID	Neurogenesis, Neurite growth, Translation, Synaptogenesis
Reelin	*RELN*	2	ASD, DD, EP, ID	Neuron migration, Neurite growth
Shank1/2/3	*SHANK1/2/3*	1S	ASD, BPD, DD, EP, ID, SCZ	Synaptogensis, Synaptic plasticit
Synapsin 1/2/3	*SYN1/2/3*	4	ASD, EP, ID	Synaptogenesis, Synaptic Function
Thousand-and-one amino acid kinase 2	*TAOK2*	N/A	ASD	Neurite growth
T-Brain-1	*TBR1*	1	ASD, ADHD, DD, EP, ID	Neuron migration
Tuberous sclerosis 1	*TSC1*	S	ASD, DD, ID	Neurite growth, Synaptogenesis, Synaptic plasticity
Tuberous sclerosis 2	*TSC2*	S	ASD, DD, ID, EP	Neurite growth, Synaptogenesis, Synaptic plasticity
Ubiquitin protein ligase E3A	*UBE3A*	3S	ASD, DO, EP, ID	Neurite growth, Synaptic plasticity
WD repeat and FYVE domain containing 3	*WDFY3*	3	ASD	Neurogenesis
δ-catenin	*CTNND2*	2	ASD	Neurite growth

*Summary of the genes reviewed including their associated disorders and the developmental processes they affect. The SFARI score = **1**, high confidence; **2**, strong candidate; **3**, suggestive evidence; **4**, minimal evidence; **5**, hypothesized but untested; **S**, mutations are associated with a high degree of increased risk and are frequently associated with additional features not required for an ASD diagnosis; **N/A**, not applicable; ASD, Autism Spectrum Disorder; ADHD, Attention Deficit Hyperactivity Disorder; DD, Developmental Delay; ID, Intellectual Disability; EP, Epilepsy; OCD, Obsessive Compulsive Disorder; SCZ, Schizophrenia; TS, Tourette’s Syndrome*.

## Alterations in Neurogenesis and Neuron Migration in ASD

### Increased Neuronal Proliferation and Macrocephaly in ASD

A growing body of literature has provided strong evidence that subsets of individuals with ASD show aberrant brain growth patterns. In patients, cerebral size may be normal at birth but display a period of increased overgrowth and a subsequent period of decline compared to unaffected control patients. An increase in neuronal numbers in prefrontal cortex has recently been observed, indicating that excess neurogenesis may be the underlying cause for an increase in cerebral size in ASD (Courchesne et al., 2011). Additionally, children diagnosed with ASD sometimes show regions of abnormal laminar positioning of cortical projection neurons (Wegiel et al., 2010). Both the development of proper lamination and the number of cortical neurons rely on temporally controlled proliferation of neural progenitors, therefore defects in neural progenitors may underlie ASD subtypes associated with overgrowth in the developing brain.

Projection neurons comprise roughly 80% of neurons in the cortex and when taking into account their dendrites, axons, myelin and synapses, they indeed could contribute to an increase in both the gray and white matter volumes in adolescents with ASD. Indeed, support for this theory has recently been shown with the comparison of neuron numbers in postmortem tissue from ASD and normal adolescents, with a surprising 67% more neurons detected in the prefrontal cortex of ASD patients (Courchesne et al., 2011). An aberrant increase in neuron numbers during prenatal neurodevelopment supports a role for a disruption of neurogenesis, which occurs mostly during gestational weeks 7–20 in humans, which coincides with the end of the embryonic period and early fetal period. Because new cell production is estimated to outnumber cell elimination by at least 100-fold in the developing brain, the role of cell death may play less of a role in macrocephaly (Rakic and Zecevic, 2000). It is important to note that programmed cell death can eliminate up to 50% of particular neuron subtypes (De Zio et al., 2005; Yamaguchi and Miura, 2015), suggesting that a shift in the balance between neuron generation and elimination could play a role in macrocephaly.

Radial glia cells must undergo a regulated series of symmetric divisions to generate more radial glia, and asymmetric divisions, to produce intermediate progenitors and post-mitotic neurons. Recently born projection neurons, principally produced via symmetric divisions of intermediate progenitor cells in the sub-ventricular zone (SVZ), will migrate in an “inside-out” fashion to establish the six layered neocortex. This mode of migration forms deeper layer neurons first, followed by later born neurons that migrate to form the outer cortical layers (Gupta et al., 2002; Nadarajah and Parnavelas, 2002; Kriegstein and Noctor, 2004). Thus, alterations in neurogenesis could lead to changes in total number neuron numbers yielded from the progenitor populations, and/or in the overall laminar structure. For example, a study identified abnormalities in lamination of the neocortex and excess neuron numbers in seven out of eight ASD cases that they examined, via magnetic resonance imaging (MRI) and postmortem histology (Hutsler et al., 2007). Cerebral dysplasia in multiple regions has been reported in ASD cases, indicative of dysregulated neurogenesis, neuronal migration and/or maturation (Wegiel et al., 2010). Additionally, Stoner et al. (2014) identified regions of disorganized cortical lamination in ten out of the eleven ASD cases they examined. These studies have provided strong evidence to support the theory of dysregulated proliferation from neural progenitors as an underlying pathology associated with macrocephaly and cortical lamination defects in ASD. Although astrocytes are generated from the same progenitor pool as neurons, the underlying cause of macrocephaly may be largely due to increased numbers of cortical projection neurons, as an associated increase in glia cells is not observed (Courchesne et al., 2011; Morgan et al., 2014). Additionally, data suggests that the cerebral white matter, which contains glia and the myelinated projections from cortical neurons, is not increased in ASD patients with macrocephaly; however, reports do indicate delayed maturation and a compromised integrity of the white matter (Hazlett et al., 2005; Friedman et al., 2006; Bakhtiari et al., 2012). These findings suggest that a preferential up-regulation in neuronal differentiation and proliferation play a major role in ASD.

The phosphatase and tensin homolog (*PTEN*) gene was the first gene to be clearly associated with macrocephaly in ASD (Goffin et al., 2001; Butler et al., 2005; Buxbaum et al., 2007; Varga et al., 2009). Pten was originally identified as a tumor suppressor and key negative regulator of phosphatidylinositol 3-kinase (PI3K) signaling, with Pten mutations found in multiple cancers (Zhao et al., 2004). In Pten-deficient mouse models, enhanced levels of phosphatidylinositol (3,4,5)-trisphosphate (PIP3) lead to activation of protein kinase B (AKT) and downstream mammalian target of rapamycin (mTOR). The mTOR pathway is well known for its ability to regulate cell growth and proliferation and consistently Pten-deficient animals show neuronal over-growth, brain enlargement, seizures and premature death (Kwon et al., 2006; Ogawa et al., 2007; Garcia-Junco-Clemente and Golshani, 2014).

*CHD8* has emerged as a key ASD-linked gene. Strikingly, 80% of individuals with mutations in *CHD8* alleles display macrocephaly, which composes a much higher percentage of total macrocephaly cases in ASD patients without *CHD8* mutations (Bernier et al., 2014). Using a mouse model, Katayama et al. (2016) have shown increased brain weight, mirroring the macrocephaly observed in humans. Using transcriptome analysis of the entire brain, the authors concluded that major targets of Chd8 were genes regulated by the RE-1 silencing transcription factor (REST), which is a neuronal transcriptional repressor (Katayama et al., 2016).

WD repeat and FYVE domain containing 3 (*WDFY3*) was identified through surveys of *de novo* variants linked to ASD (Iossifov et al., 2012, 2014), which is implicated in macrocephaly and altered neural progenitor proliferation. Wdfy3 is a scaffold protein, involved in macroautophagy of large aggregation-prone proteins (Filimonenko et al., 2010). Decreased Wdfy3 expression in mice produces pronounced effects on neuronal proliferation and migration. Wdfy3 mutant mice also display macrocephaly resulting from a shift of radial glia divisions from asymmetric to symmetric (Orosco et al., 2014). This change in division ultimately produces greater numbers of neuronal progenitors and therefore brain size. Wdfy3’s function in regulating cellular division is unclear, however expression studies have shown that it is up-regulated during cellular division and Wdfy3 plays a role in autophagy and regulation of proteins that control the cell cycle, ultimately resulting in a shortened cell cycle in Wdfy3 mutant mice (Orosco et al., 2014), and that additionally, because progenitor expansion and neurogenesis initiates anterolaterally and concludes posteromedially (Caviness et al., 2009), Wdfy3 mutant mice show a more pronounced affect in the anterolateral areas. This finding is in line with MRI performed in ASD adolescents, wherein temporal and frontal cortical areas showed the largest size increases (Carper et al., 2002; Hazlett et al., 2005; Schumann et al., 2010). Notably, these region-specific changes of cerebral growth could be associated with key behavioral symptoms observed in ASD. In humans, areas such as the superior temporal sulcus and parts of the prefrontal and temporal cortex, which are key regions involved in reward and reinforcement pathways as well as social and emotional pathways are susceptible in ASD patients (Pelphrey and Carter, 2008; Redcay, 2008; Gotts et al., 2012; Gasquoine, 2014).

The association of macrocephaly with clinical phenotypes in autism has been characterized in an inconsistent manner and previous studies have indicated higher levels of cognitive function in patients with macrocephaly and ASD compared to normal controls (Courchesne and Pierce, 2005; Sacco et al., 2007). An increase in head circumference has been shown in ASD patients with special capabilities, compared to those without them (Ben-Itzchak et al., 2013a). However, additional studies have not discovered similar correlations with increased head circumferences and special abilities (Gillberg and de Souza, 2002; Ben-Itzchak and Zachor, 2007).

### Genes Associated with Mitotic Dysregulation of Neural Progenitors and Microcephaly in ASD

Microcephaly has not been studied as thoroughly as macrocephaly in ASD patients. The reports have indicated an increased prevalence of microcephaly in ASD, with up to 20% of cases, in comparison to 3% reported in the general population. Additionally, microcephaly is more frequent in individuals with ID and higher ASD severity (Fombonne et al., 1999; Cody et al., 2002; Miles et al., 2005; Ben-Itzchak et al., 2013b).

Autosomal recessive primary microcephaly (MCPH) is a condition that displays with significantly reduced head circumference that develops during the prenatal period (Tunca et al., 2006). The development of the forebrain is prominently affected in this form of microcephaly ultimately results in ID (Roberts et al., 2002; Bond et al., 2003). Disruption in genes encoding proteins that localize to the centrosome are known to result in MCPH (Kaindl et al., 2010), including Microcephalin 1 (*MCPH1*). *MCPH1* is a gene expressed during fetal development and mutations in *MCPH1* produce microcephaly (Jackson et al., 1998, 2002). Studies have found rare variants in the *MCPH1* gene that are linked to ASD (Ozgen et al., 2009; Neale et al., 2012), and play a role in DNA-damage repair, chromosome condensation, and the regulation of DNA damage genes (Thornton and Woods, 2009; Richards et al., 2010). Mechanistically, it has been shown that Mcph1 regulates neuroprogenitor division by coupling centrosomal maturation and mitotic spindle orientation with mitotic entry (Gruber et al., 2011).

Abnormal Spindle Primary Microcephaly (*ASPM*, *MCPH5*) is involved in orientation of the mitotic spindle, regulation of mitosis and cytokinesis (Fish et al., 2006; Passemard et al., 2009, 2016; Higgins et al., 2010). Mutant Aspm mice have mild microcephaly without aberrant increase in cell death, suggesting that disruptions in the proliferation of embryonic neuronal progenitor cells underlie MCPH (Pulvers et al., 2010). Additionally, Aspm positively regulates Wnt signaling (Major et al., 2008), and over-expression of β-catenin, a positive transducer of the Wnt pathway, can rescue neurogenesis deficits in mice (Buchman et al., 2011).

Mutations in WD Repeat Domain 62 (*WDR62*) are associated with microcephaly and other cortical abnormalities in humans (Bilgüvar et al., 2010; Yu et al., 2010). Wdr62 deficient mice have reduced brain size due to decreased neural progenitor cell population cells show mitotic spindle instability, mitotic arrest and cell death after loss of Wdr62 expression (Chen et al., 2014). Mutations or loss of Wdr62 expression therefore leads to mitotic delay and death of neural progenitor cells, thereby resulting in microcephaly.

### Alterations in Neuron Migration in ASD

#### Excitatory Projection Neuron Migration

**Figure 1:**
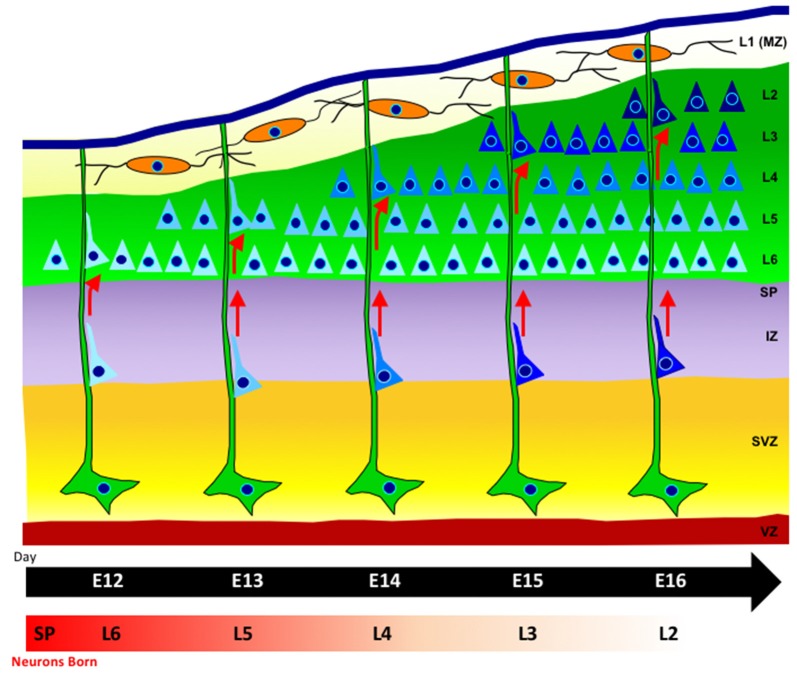
Radial-glia guided neuronal migration. Radial glia cells (green), extend long basal process to the pial surface, with their somas located in the ventricular zone (VZ). Neurons (blue) are born in the VZ and migrate along radial glia fibers. The cortical plate is formed in an inside-out fashion such that later-born neurons that will reside in the upper layers pass through earlier-born neurons in deeper layers (lighter blue shading). The marginal zone contains horizontally-oriented Cajal-Retzius cells (orange) which release the extracellular signaling glycoprotein Reelin.

The time of birth of neocortical neurons, as well as their proper movement from the proliferative zone, dictates their final position within the layers of the cerebral cortex (Angevine and Sidman, 1961). Neurons born later during neurogenesis will ultimately reach the superficial layers above earlier born neurons, due to an “inside-out” mode of neuronal migration. Initially, multipolar neurons will migrate via cellular locomotion. As neurons continue into the neocortex, they adhere to and migrate along radial glia, which provide aid in the direction of their migration (Rakic, 1972; Hatten, 1999; Kriegstein and Noctor, 2004; Ayala et al., 2007). Neurons adopt a bipolar morphology as they move along radial glia and detach upon reaching their proper laminar position (Figure 1). Additionally, daughter neurons in different cortical layers that were generated from the same mother progenitor cell will align radially into highly connected mini-columnar structures, most likely constituting a functional unit in the brain (Gao et al., 2013).

Studies have shown that defects in neuronal migration are associated with changes in neuron density and soma size, irregular minicolumns and heterotopias (mis-localized neurons; DiCicco-Bloom et al., 2006; Uppal et al., 2014; Chen et al., 2015; Reiner et al., 2016). In humans for example, two separate studies have demonstrated evidence of aberrant cell patterning in the boundary of cortical gray–white matter, suggesting defects in neuronal migration (Hutsler et al., 2007; Avino and Hutsler, 2010). In another study, an abnormal lamination of neurons, but not glia, was detected in the cortex of adolescent ASD brain samples (Stoner et al., 2014).

Reelin is one of the best known regulators involved in neuronal migration (Folsom and Fatemi, 2013; Sekine et al., 2014). Reelin is a glycoprotein that is released from Cajal–Retzius cells in the outer marginal zone. Reelin binds to the receptors Apoer2 and Vldlr on the cell membrane of target neurons, thereby inducing tyrosine phosphorylation of the adaptor protein Dab1, which subsequently initiates signaling cascades (Forster et al., 2006; Pardo and Eberhart, 2007). Reelin is involved in the termination of migration, proper neuronal layering, as well as transition to a bipolar morphology prior to neuronal migration along radial glia. Studies using postmortem human samples suggest aberrant Reelin signaling in ASD patients (Persico et al., 2001; Bonora et al., 2003; Fatemi et al., 2005). Reelin mutations in humans produce disruptions in neuron migration and connectivity, lissencephaly and cerebellar hypoplasia (Hong et al., 2000). In the well-studied Reelin knockout (KO) mice, neuronal migration abnormalities result in an inversion of cortical layering (Falconer, 1951; D’Arcangelo et al., 1995; Hirotsune et al., 1995). Behavioral studies in Reelin KO mice show aggressive behavior, an abnormal gait, social aggression, and impairments in learning and memory (Salinger et al., 2003). T-Box Brain Protein 1 (*TBR1*) encodes a brain-specific T-box transcription factor, which plays an important role in neurodevelopment and migration and has been identified as a causative gene for ASD (Neale et al., 2012; Deriziotis et al., 2014; De Rubeis et al., 2014). Tbr1 is involved in the differentiation of neurons from intermediate progenitors early in the development of the neocortex (Dwyer and O’Leary, 2001; Han et al., 2011; Willsey et al., 2013). Tbr1 regulates the expression of many ASD-linked genes (Chuang et al., 2015), including the activation of Autism Susceptibility Candidate 2 (*AUTS2*), which has been identified as an ASD and ID associated gene (Bedogni et al., 2010; Srinivasan et al., 2012). Interestingly, *TBR1* KO mice do not express subplate, layer 6 or Cajal-Retzius cell markers and show a large decrease in Reelin expression, however upper cortical layers are typically normal (Hevner et al., 2001).

Multiple mutations within *AUTS2* have been identified in patients, strongly linking it as a causative factor for ASD (Sultana et al., 2002; Huang et al., 2010; Pinto et al., 2010; Talkowski et al., 2012; Cheng et al., 2013; Liu et al., 2015). The Auts2 protein is predominantly nuclear localized and evidence suggests a role for Auts2 in regulating gene expression during brain development (Bedogni et al., 2010; Srinivasan et al., 2012; Gao et al., 2014). Binding of Auts2 with polycomb repressive complex 1 (PRC1) inhibits PRC1 activity leading to activation of gene transcription (Gao et al., 2014). In addition to the nuclear component, Auts2 protein is also found in the cytoplasm, where it lays a role in regulating cortical neuronal migration and neurite growth (Hori et al., 2014).

In a cohort of Amish children, mutations within contactin-associated protein-like 2 gene (*CNTNAP2*) were found to be implicated in ASD and epilepsy (Strauss et al., 2006). CNTNAP2 is a scaffolding protein that’s part of the Neurexin family, whose members have previously been associated with multiple ASD-linked proteins (Jamain et al., 2003; Comoletti et al., 2004). Ectopic neurons were identified in patients with CNTNAP2 mutations, suggesting its involvement in the regulation of neuronal migration (Strauss et al., 2006). Additionally, CNTNAP2 mutations are linked to ADHD as well as epilepsy and seizures, conditions that are commonly co-morbid with ASD (Jackman et al., 2009; Elia et al., 2010; Mefford et al., 2010). Additionally, disruptions in CNTNAP2 are associated with impairments in sociability and language processing (Whalley et al., 2011; Toma et al., 2013; Condro and White, 2014). CNTNAP2 KO mice show an abnormal localization of neurons in the corpus callosum and mis-localization of the Cux1-positive upper layer neurons into the deeper layers, V–VI (Peñagarikano et al., 2011). CNTNAP2 is part of a neuron-glia adhesion complex with contactin 2, therefore it may play a key role in radial glia-guided neuronal migration (Poliak et al., 1999; Denaxa et al., 2001).

Astrotactin 1 (*ASTN1*) is a neuronal cell surface antigen that regulates neuron-glia interactions that plays a major role in neuron migration (Edmondson et al., 1988; Zheng et al., 1996). Astn1 KO mice have slower cerebellar granule cell migration, aberrant Purkinje cell morphology, decreased glia-neuron interactions and impairments in coordination compared to normal mice (Adams et al., 2002). Astn1 interacts with Astn2 and can regulate its expression at membrane surface, which ultimately regulates neuron-glia adhesion during migration along radial glia (Wilson et al., 2010). Genome wide association studies identified *ASTN2* as an ASD candidate gene (Lesch et al., 2008). Patients with *ASTN2* deletions are often classified with ASD or other co-morbid diagnoses such as ADHD, obsessive compulsive disorder and delayed language development (Lionel et al., 2014).

NudE nuclear distribution E homolog (*NDE1*) is involved with the regulation of neuron proliferation, migration, and intracellular transport as part of the Lis1/Nde/Ndel1/cytoplasmic dynein complex (Feng et al., 2000; Kitagawa et al., 2000; Niethammer et al., 2000; Sasaki et al., 2000; McKenney et al., 2010). Lissencephaly 1 *(LIS1)* was the first gene identified with an involvement in disrupted neuron migration (Reiner et al., 1993) via its interactions with cytoplasmic dynein, Nde1, Ndel1 and cytoplasmic linker protein 170 (CLIP-170; Reiner, 2000; Coquelle et al., 2002). Multiple studies have shown a major role for Lis1 in regulating neuronal migration (Cahana et al., 2001; Tsai et al., 2007; Hippenmeyer et al., 2010) and a cross between an Nde1 KO mouse with Lis1 heterozygous mouse produced a severe disruption of the morphology of the VZ progenitors and radial glia, as well as a significant decrease in brain size (Pawlisz et al., 2008). This was shown to be regulated via stabilization of the dystrophin/dystroglycan glycoprotein complex (Pawlisz and Feng, 2011) and an alteration of the MAPK scaffold protein Kinase Suppressor of Ras (KSR), subsequently producing hyperactivation of MAPK/ERK pathway (Lanctot et al., 2013). In support of these findings, additional studies have shown that dysregulation of the MAPK/ERK pathway affects social behavior in mice and produces ASD phenotypes, while multiple links between ASD and hyperactivation of the Ras signaling have been reported (Levitt and Campbell, 2009; Rauen et al., 2010; Crepel et al., 2011; Faridar et al., 2014).

#### Inhibitory Neuron Migration

Interneurons, which are primarily inhibitory, utilize tangential migration to migrate into the neocortex and across the plane of radial glia fibers (Lavdas et al., 1999; Marín and Rubenstein, 2001; Nery et al., 2002; Kriegstein and Noctor, 2004; Ayala et al., 2007). Upon reaching the cerebral cortex, interneurons will migrate along radial glia fibers to their final location (de Carlos et al., 1996; Wichterle et al., 2001; Polleux et al., 2002; Poluch and Juliano, 2007). Therefore, when radial glia-mediated migration becomes disrupted, interneurons will also be become mis-localized.

Distal-less homeobox (*Dlx*) genes are part of the homeodomain transcription factor family which is related to the *Drosophila* distal-less (*Dll*) gene (Panganiban and Rubenstein, 2002). Dlx1/2 are found largely in the ganglionic eminences (GE) of the developing brain and play an important role in regulating the migration of inhibitory interneurons from the medial GE into the cortex. Dlx1 KO mice exhibit a decrease in GABAergic neurons and present with epilepsy, a commonly observed pathology in ASD patients (Cobos et al., 2005). *Dlx1*/*Dlx2* double KO mice show major aberrations in the migratory stream of GABAergic neurons and also an accumulation of neuronal precursor cells in the GE (Anderson et al., 1997; Ghanem et al., 2007). *Arx*, an X-linked homeobox gene and immediate downstream target of Dlx, may regulate Dlx’s role in tangential neuronal migration (Colasante et al., 2008). Patients identified with mutations in *Arx* display ID, autistic features and epilepsy (Strømme et al., 2002; Turner et al., 2002).

## Alterations in Neurite Growth and Spine Formation in ASD

Neurons are highly specialized cells with distinct morphologies comprised of three distinct sections: the soma which contains the nucleus and the majority of the cellular organelles; a long axonal process to transmit information; and a complex dendritic arbor to receive information from neighboring neurons. The dendrites are highly branched and elaborate and therefore occupy a large area within neural tissues (Tahirovic and Bradke, 2009).

Dendrite growth can be viewed as discreet steps. After birth from neural progenitors, neurons start as a simple round soma and must first undergo cellular polarization. Neurons first adopt a multipolar morphology with the extension of minor neurites. The movement of migrating neurons is achieved through the formation, maintenance and constant transformation of microtubules in response to extracellular cues and intracellular polarity signals. During migration, the cell first extends a leading process. Stabilization of a single neurite is required for newly generated neurons to exit the multipolar stage to enter the cortical plate. This stabilization ultimately results in formation of the leading process while the trailing process eventually develops into the future axon (Shim et al., 2008; Witte and Bradke, 2008). Next, in order to move forward, the nucleus undergoes a translocation into the stabilized leading neurite. During this process, termed nucleokinesis, the attachment of microtubules from the centrosome to the nuclear envelope exerts a traction force, pulling the nucleus into the leading neurite attached to a radial glia process (Bellion et al., 2005; Tahirovic and Bradke, 2009).

**Figure 2:**
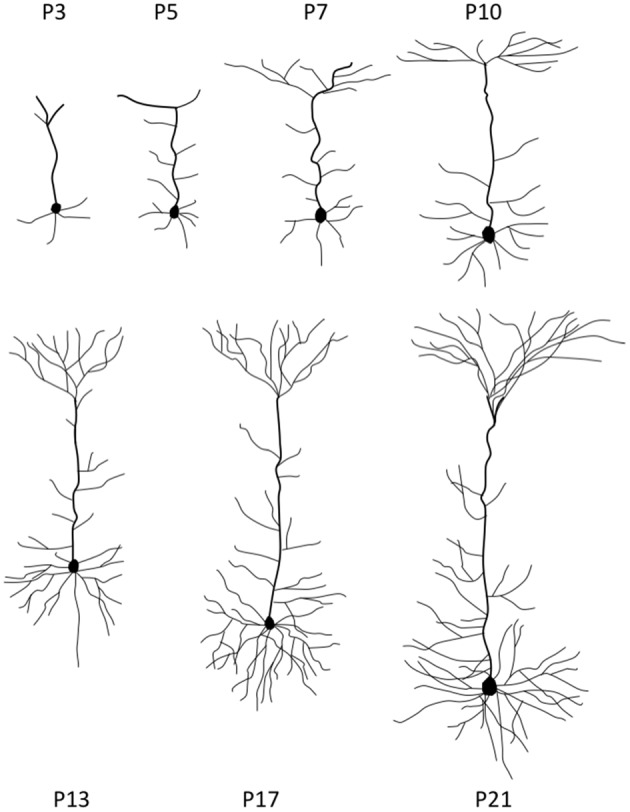
Stages of neurite growth. Representative neocortical mouse pyramidal neuron morphologies at different early postnatal time points during development. P = postnatal.

Once neurons reached their destined cortical layers, substantial dendritic outgrowth is undertaken to form dendritic arbors characteristic of a neuron subtype. At this stage, neurons are tasked with the processes of growing each branch to the correct size, initiating a new branch at the right site to complete a specific branching pattern, and directing each branch’s growth into an appropriate spatial location (Tahirovic and Bradke, 2009; Jan and Jan, 2010). The early postnatal stages of neurite growth are depicted in Figure 2.

Dendrites are vastly different from axons in their ultimate morphology, function and developmental processes. In mammalian neurons, a notable distinction is the manner in which the microtubules are organized. Initially all neurites contain microtubules oriented with their plus-end localized distally from the soma. The neurite specified to become the axon will maintain this distal plus-end orientation, while neurites that become dendrites will adopt a mixed orientation (Baas et al., 1988, 1989; Burton, 1988). Additionally, in comparison to axons, dendrites have an enrichment of cellular organelles that are transported with the aid of microtubule motors like dynein, such as Golgi outposts and mitochondria, which are utilized to supply the necessary for cellular materials required for growth (Horton and Ehlers, 2004; Horton et al., 2005; Kapitein et al., 2010).

The morphology of the dendritic arbor is largely developed early during the embryonic period; however, dendrites are highly dynamic and they maintain the overall morphology with various mechanisms into adulthood. Disruptions in dendritic growth, or breakdown of the mechanisms to maintain their morphology can be deleterious, resulting in aberrant network function. Abnormalities in neuronal connectivity between the higher-order association areas have been considered one of the major defects in ASD (Geschwind and Levitt, 2007). MRI studies have shown both large and small changes in connectivity of neuronal networks; however, the neurobiological basis for this disconnectivity remains to be fully elucidated (Casanova and Casanova, 2014; Maximo et al., 2014). A large number of autism studies have focused on dendritic development, although mostly relating to spine morphology and synaptic function, which will be discussed in detail below (Persico and Bourgeron, 2006; Kelleher and Bear, 2008; Bourgeron, 2009). However, the current body of research suggests that this is not the only morphological defect, as children with autism are frequently identified with large-scale anatomical abnormalities, suggesting dysregulation in dendritic growth and development. For example, both macrocephaly and microcephaly are identified in ASD adolescents, with 15% of ASD patients presenting with macrocephaly, while 20% display microcephaly (Lainhart et al., 1997; Fombonne et al., 1999; Cody et al., 2002; Pardo and Eberhart, 2007).

One of the proposed causes leading to macrocephaly has been suggested to be a result of increased dendrite number and size, which may be a product of excessive dendrite arborization, and/or decreased dendrite pruning (Jan and Jan, 2010). Other factors can play a role in macrocephaly, such as increased numbers of neurons and glia, however the number of dendrites may better explain certain forms of macrocephaly. Brain volumes in infants diagnosed with ASD are typically normal, however they display aberrant overgrowth as development progresses. This later onset of increased brain volume should not result from an increase in neuronal number, as the largest areas of the brain have mostly completed neurogenesis prior to birth, except for areas like the hippocampus where smaller amounts of neurogenesis occur later in life (Ming and Song, 2005; Zhao et al., 2008). The timing of increase brain size can be explained by aberrant dendrite growth and branching, which largely occurs postnatally. In humans, neurons grow and continue to elaborate their dendritic arbors, axons and form new synaptic connections until the age of five, with experience-based remodeling of synapses until 20 years of age (Stiles and Jernigan, 2010; Tau and Peterson, 2010; Pescosolido et al., 2012). Dendrites continue to extensively grow after birth, in an activity-dependent manner. Therefore, a large contribution to the increase in brain size postnatally likely results from increased dendritic growth (Redmond et al., 2002).

Previous genetics studies have described multiple high-risk genes for autism that play roles in diverse functions, including synaptic connectivity and synapse function, dendritic and axonal growth, trafficking, transcription and translation (Volders et al., 2011; de Anda et al., 2012; Miao et al., 2013; Bakos et al., 2015). Recently, estimates have suggested that 88% of the genes that are considered to be high-risk for ASD play a role in early neurodevelopmental functions such as neurogenesis and differentiation of neuroblasts. Importantly, ~80% of these genes are involved in later phases of neurodevelopment and regulate processes involved in neurite growth and synapse formation (Casanova and Casanova, 2014).

Dendrites from an individual neuron can have a thousand or more spines, with each spine forming an excitatory synaptic connection upon maturing. Spines and synapses are produced in excess numbers during development, but synaptic numbers are later fine-tuned through activity-dependent stabilization or elimination (Changeux and Danchin, 1976). Spines are highly dynamic and undergo constant turnover and morphological plasticity with a dependency on both developmental stage and activity.

Dendritic spines are typically classified as thin, stubby and mushroom, with the latter considered more mature. Mushroom and stubby morphologies are more permanent and form strong excitatory connections (Trachtenberg et al., 2002; Kasai et al., 2003). Alternatively, thin spines are highly dynamic, shorter lasting, and form weak synaptic connections or no connection at all. Within the spine head, actin becomes enriched helps play a key role in spine formation and structural dynamics (Chazeau and Giannone, 2016). Previous studies have shown that spine formation maintenance are a major cellular processes affected in ASD (Kelleher and Bear, 2008; Bourgeron, 2009; Phillips and Pozzo-Miller, 2015). Using Golgi staining, postmortem analysis of cortical neurons from ASD brain samples showed an increase in dendritic spine density compared to normal patients (Hutsler and Zhang, 2010). Some of the high-risk autism genes implicated in dendritic growth and branching spine formation and synapse maturation are described below.

Methyl CpG binding protein 2 (*MECP2*) is an X-linked gene that codes for a protein that functions as a transcriptional repressor and an activator (Chahrour et al., 2008). Mutations in MeCP2 were initially linked to Rett syndrome (RTT), a neurodevelopmental condition that presents with motor and speech impairments, cognitive deficits and autism (Amir et al., 1999). RTT is typically caused by loss-of-function mutations in MeCP2; however, there are rare cases that are also caused by MeCP2 duplications.

Using Golgi staining in brain slices from MeCP2 KO mice, reductions in dendritic growth and branching have previously been reported in both apical and basal arbors of motor cortical neurons (Kishi and Macklis, 2004; Stuss et al., 2012). Additionally, to investigate MeCP2 duplication, mice over-expressing the human MeCP2 gene have shown excessive dendritic branching, indicating that MeCP2 over-expression can induce dendritic overgrowth (Jiang et al., 2013). It is possible that in subtypes of neurons MeCP2 can act as a repressor while acting as an activator in others, or it can change roles at different periods in neuronal development. For instance, MeCP2 can activate genes involved in early dendrite growth and repress genes later during dendritic remodeling. An important aspect to these findings is that MeCP2 overexpression affects the dendrites, having no impact on axon growth, suggesting a defined role for MeCP2 in dendritic development. Recent work has provided evidence for the role MeCP2 plays in dendritic development, showing that MeCP2 also regulates the expression of genes post-transcriptionally. These findings show that MeCP2 regulates microRNA (miRNA) processing via a direct interaction with DiGeorge syndrome critical region 8 (DGCR8), which plays a role in mediating the genesis of miRNAs thought to regulate dendrite morphogenesis (Gregory et al., 2004; Cheng et al., 2014).

RTT animal models also show changes in excitatory hippocampal synapse numbers. The loss of the X-linked RTT protein MeCP2 has also been show to result in abnormal dendritic spine morphology and a decrease in spine density (Zhou et al., 2006; Chapleau et al., 2012; Stuss et al., 2012). Additionally, MeCP2 over-expression produces dendritic overgrowth in mice and these animals show a greater rate of spine turnover, with a bias toward spine removal (Jugloff et al., 2005; Jiang et al., 2013). Taken together, MeCP2, the established factor underlying RTT with autism, has a clear role in dendritic morphogenesis and synapse formation, both of which should play a major function in the cognitive impairments seen in RTT patients.

Fragile X mental retardation gene 1 (*FMR1*) is the gene underlying the disorder Fragile X syndrome (FXS), which results in ID with 15%–30% of patients also displaying ASD phenotypes (Krawczun et al., 1985; Persico and Bourgeron, 2006; Kelleher and Bear, 2008; Santoro et al., 2012). FXS is usually results from an expansion of a CGG triplet in the 5′-UTR region of the FMR1 gene, however a few missense mutations and deletions have been identified (Santoro et al., 2012). Fragile X mental retardation protein (FMRP), the *FMR1* gene product, plays a key role in negatively regulating translation, especially local translation at the synapse (Santoro et al., 2012). In terms of FMRP’s role in dendritic growth, mouse studies have shown somewhat contradictory results. In visual cortex pyramidal neurons of *FMR1* KO mice, one study has shown defects in dendritic spines, with no observable changes in dendritic morphology (Irwin et al., 2002). Conversely, multiple other studies have shown that FMRP is critical for dendritic growth and branching. In *FMR1* KO mice, visual cortex pyramidal neurons show reduced basal dendrite length and branching (Restivo et al., 2005). Using neural stem cells isolated from *FMR1* KO mice, or from postmortem tissues of FXS human fetuses, differentiated neurons showed fewer and less complex neurites with smaller somas (Castrén et al., 2005). An important caveat to these studies was that they involved loss-of-function or deletion of FMRP; however, FXS in humans is rarely caused by deletions or missense mutations in the *FMR1* gene, but rather via an expansion of the CGG triplet repeat. This has been addressed in studies using transgenic mice with a FXS knock-in mutation consisting of 120–140 CGG repeats. Indeed, these animals display significantly impaired dendritic morphogenesis in addition to alterations in dendritic spine density and morphology (Berman et al., 2012). The studies from *Drosophila* have elucidated the mechanistic function of FMRP in dendritic growth and have shown that FMRP is involved with transportation of the mRNA for Ras-related C3 botulinum toxin substrate 1 (Rac1), a GTPase. In agreement with this, Rac1 was found to bind FMRP and affect dendrite arborization, indicating that FMRP’s role in dendrite morphogenesis could in part be through its interaction with Rac1 (Lee et al., 2003). These findings strongly support a role for FMRP in dendritic arbor morphogenesis.

FMRP is localized within dendrites and in addition to abnormal dendritic growth and branching, brains from FXS patients display an immature synaptic phenotype (Rudelli et al., 1985). FXS patients have an increased spine density on both apical and basal dendrites in neocortex and more spines characterized by an immature morphology. Additionally, Golgi studies from human FXS patients reveal a significant increase in long spines with less shorter spines compared to controls in multiple cortical areas (Hinton et al., 1991; Irwin et al., 2001). In *FMR1* KO mouse models, spine phenotypes correlate with those observed in humans with FXS as these mice have an increase in longer spines and a corresponding decrease in shorter spines. Additionally, dendritic spines in FXS mice display a more immature morphology with fewer mushroom and stubby spines present (Irwin et al., 2001, 2002; McKinney et al., 2005). In addition, changes in synaptic proteins such as postsynaptic density-95 kDa (PSD-95) have been observed in FXS (Ifrim et al., 2015).

Although FMRP’s role in impaired spine formation and morphology in FXS has not been completely elucidated, FMRP has been identified to interact with multiple proteins that are linked to dendritic and spine regulation. Cytoplasmic FMRP-interacting protein 1 (CYFIP1), a binding partner of FMRP, is a protein that has recently generated interest as its genetic locus is chr15q11.2, a susceptibility area in ASD. When bound to FMRP, CYFIP1 can inhibit translation and regulates actin dynamics. This can therefore regulate the growth and removal, as well as the labiality and morphology of spines (De Rubeis et al., 2013). A down-regulation of CYFIP1 mRNA has been detected in subgroups of FXS patients with ASD (Nowicki et al., 2007). Conversely, over-expression of CYFIP1 results in higher dendritic complexity *in vitro*, while neurons that are haploinsufficient for CYFIP1 show decreased dendritic complexity and an increase in the relative levels of immature to mature dendritic spines (Pathania et al., 2014).

Pten is a phosphatase that de-phosphorylates PIP3, which serves to inhibit PI3K/AKT/mTOR signaling (Kwon et al., 2006; Ogawa et al., 2007; Garcia-Junco-Clemente and Golshani, 2014). Mice lacking Pten selectively in the central nervous system show an increase in activation of the AKT/mTOR/S6K pathway, an increase in neuron size, macrocephaly, as well as decreased and disorganized dendritic and axonal growth (Kwon et al., 2006). Pten KO during early development, as well as in adult mice disrupts neuron morphogenesis, suggesting that Pten plays an important role in dendritic growth and maintenance in adulthood in addition to its function during early neurodevelopment (Kwon et al., 2006; Chow et al., 2009). Therefore, Pten, a protein with a clear link to autism, has a definitive role in dendrite morphogenesis.

*TSC1* and *TSC2* are tumor-suppressing genes that have been linked to brain tumors in tuberous sclerosis complex (TSC; Huang and Manning, 2008). However, their involvement in neurodevelopmental disorders including epilepsy, autism and ID has become increasingly studied (Kwiatkowski and Manning, 2005; Persico and Bourgeron, 2006). In pheochromocytoma 12 (PC12) cells, an inducible neuron-like cell line, transfection of *TSC1* antisense oligonucleotides was shown to increase neurite outgrowth through RhoA activation, while knockdown of Tsc2 decreased neurite growth (Floricel et al., 2007). In another study, overexpression of Tsc1/Tsc2 was found to impair axon formation. Knockdown of Tsc1/Tsc2 *in vitro* induced multiple axons, while genetic deletion *in vivo* in the mouse induced ectopic axons (Choi et al., 2008). There is strong evidence that TSC genes are linked to ASD risk and play a major role in neurite growth, therefore it will be important to further elucidate their mechanistic role in neuron morphogenesis and ASD in future research.

Mammalian target of rapamycin (mTOR is a serine/threonine kinase that regulates cellular growth and is induced by growth factors and environmental cues (Laplante and Sabatini, 2012). Via regulation of the cytoskeleton, mTOR plays an important function in regulating dendritic outgrowth, and proteins known to inhibit mTOR signaling have been associated with aberrant dendrite and spine development in ASD (Thomanetz et al., 2013; Skalecka et al., 2016), including Pten and Tsc 1/2 (Weston et al., 2014). Pten serves to inhibit the PI3K/AKT/mTOR signaling pathway, thereby affecting growth and protein translation, and Pten mutations have been discovered in multiple individuals diagnosed with ASD. Additionally, mice carrying PTEN mutations or genetic deletions show impaired social interactions and increased sensory responses. Loss of Pten produces an increase in dendritic growth, synaptic connectivity and disorganized dendritic and axonal processes (Kwon et al., 2006; Orloff et al., 2013). Conversely, knockdown of Pten in the amygdala decreases spine density, with an increase in mushroom spines and decrease in thin protrusions (Haws et al., 2014). Additionally, it has been previously shown that the TSC pathway plays an important role in regulating synaptic function, and in hippocampal neurons, loss of Tsc 2 expression produces an expansion of neuronal somas as well as spines (Tavazoie et al., 2005). These findings provide strong evidence that mTOR, and its interacting proteins, are involved in regulating both dendritic and spine development in ASD.

Neurofibromatosis is a condition in which tumors grow in the nervous system. Mutations in Neurofibromatosis-1 (*NF1*) produce neurofibromas and between 30% and 65% of children with *NF1* mutations display learning disabilities and display significantly higher rates of autism, suggesting a causal relationship to autism (Rosser and Packer, 2003; Garg et al., 2013). Neurofibromin, the protein product of the *NF1* gene, is a GTPase activating protein that negatively regulates the Ras signaling pathway (Costa and Silva, 2003). *NF1* conditional KO mice display impaired dendritic morphogenesis and Golgi staining from *NF1* KO mouse brains reveals shorter apical dendrites in pyramidal neurons. *NF1*’s function in dendrite morphogenesis has been shown to act through cAMP/PKA/Rho/ROCK signaling (Brown et al., 2012). RhoA plays a major role in actin dynamics and neurite growth and branching. With the inhibition of RhoA, an increase in the branching of neuronal processes is observed, and with the activation of RhoA, a decrease in the length and complexity of processes is observed (Li et al., 2000; Nakayama et al., 2000; Wong et al., 2000).

Loss of expression of *KIAA2022/*KIDLIA was previously identified by our group and others as the causative protein for severe ID and autistic behavior in multiple families (Cantagrel et al., 2004, 2009; Ishikawa et al., 2012; Van Maldergem et al., 2013; Charzewska et al., 2015; Kuroda et al., 2015). Clinical examinations of patients with KIDLIA mutations or a loss of expression, display enlarged ventricles, Virchow-Robin spaces, a thin corpus callosum and small cerebellar vermis. Additionally, strabismus has been observed in patients, with some dysmorphic features including a round face during early postnatal periods, febrile seizures, severely impaired or no language, stereotypical hand movements and delayed acquisition of motor milestones (Cantagrel et al., 2004, 2009). Interestingly, a patient with decreased expression of KIDLIA had only mild cognitive deficits with a significant delay in language acquisition as well as repetitive and stereotyped behaviors, indicating that the effects may be gene-dosage dependent (Van Maldergem et al., 2013).

With shRNA-mediated knockdown of KIDLIA in rat hippocampal neurons in culture, significant impairments in dendritic growth and branching are observed (Van Maldergem et al., 2013; Gilbert and Man, 2016). Mechanistically, we’ve recently reported that loss of KIDLIA significantly impairs actin dynamics and produces an aberrant increase in total and membrane-localized N-cadherin. N-cadherin was found to bind much greater levels of δ-catenin, thereby releasing the latter’s inhibition on downstream RhoA (Gilbert et al., 2016). Additionally, microcephaly has been reported in human patients with a loss of KIDLIA expression (Cantagrel et al., 2004; Van Maldergem et al., 2013; Kuroda et al., 2015). These findings suggest that the regulation of neuronal morphogenesis through dendrite growth and synapse formation are major underlying factors contributing to the cognitive impairments observed in patients with genetic deletions or functional mutations in KIDLIA.

Ubiquitin protein ligase E3A (*UBE3A*) is the causative gene for Angelman syndrome, a developmental disorder characterized by language impairments, ataxia, ID and hyperactivity (Williams et al., 2006). Individuals identified with Angelman syndrome are often co-diagnosed with autism (Steffenburg et al., 1996; Peters et al., 2004). In addition, Ube3A over-expression leads to autism in humans and in animal models (Bucan et al., 2009; Smith et al., 2011; Noor et al., 2015). *UBE3A* is an imprinted gene that encodes an E3 ubiquitin ligase, that typically expresses from both maternal and paternal alleles in most tissues, but is only expressed from the maternal allele in brain (Mabb et al., 2011). Previous studies have shown that Ube3A is required for normal dendritic morphogenesis in the mouse (Miao et al., 2013) and shRNA-mediated knockdown of Ube3A *in vivo* impairs dendritic growth cortical pyramidal neurons in the mouse (Miao et al., 2013). Additionally, studies in *Drosophila* strongly support a role for Ube3A in dendritic growth and branching. Both over-expression and loss of the Ube3A *Drosophila* homolog have been shown to result in decreased dendrite branching in larval sensory neurons (Lu et al., 2009). These studies suggest that Ube3A plays a significant role in dendrite growth and support that dysregulated neuron morphogenesis may underlie developmental disorders like Angelman syndrome and ASD.

Thousand-and-one amino acid kinase 2 (*TAOK2*) belongs to the family of MAP kinase kinase kinases (MAPKKK) and is located in chromosome 16, an area associated with increased ASD-risk (Weiss et al., 2008) and schizophrenia (McCarthy et al., 2009). Both Taok1 and Taok2 activate mitogen-activated protein kinase (MAPK) pathways via JNK and p38 which results in regulation of gene transcription (Chen and Cobb, 2001; Chen et al., 2003). Additionally, FMRP regulates Taok2 mRNA (Darnell et al., 2011), the protein underlying FXS, providing an additional link to its involvement in neurodevelopmental disorders. A recent study *in vivo* has shown that knockdown or overexpression of Taok2 showed opposing effects on basal dendrite development in the neocortex (de Anda et al., 2012). Specifically, primary dendrite numbers were reduced after Taok2 knockdown, while the number of primary dendrite numbers was increased with over-expression. Additionally, this effect was preferential for the basal dendrites on basal dendrites, as Taok2 knockdown produced no changes in apical dendrite morphology on the same neurons. The role of Taok2 on dendrite growth was dependent on interactions with Neuropilin 1, a membrane receptor that binds Semaphorin 3A, which leads to initiation of the JNK cascade (de Anda et al., 2012). Interestingly, this finding shows that Taok2 expression selectively affects specific dendritic areas, i.e., the basal dendrites, providing evidence that specialized molecular pathways are used for the formation of different dendritic areas.

Reelin, as discussed previously for its role in neuron migration, seems to also play a significant function in dendrite arborization in hippocampal and cortical neurons (Niu et al., 2004; Jossin and Goffinet, 2007; MacLaurin et al., 2007; Chameau et al., 2009; Hoe et al., 2009; Matsuki et al., 2010). Reelin, and its downstream signaling pathway through Vldrl/Apoer2-Dab1, serves to promote dendrite development. Reeler mice contain a loss-of-function mutation in *RELN*, and display decreased dendrite branching in the hippocampus (Niu et al., 2004). Additionally, Reelin has also been shown to affect cortical dendritic growth *in vivo* (Hoe et al., 2009) and Reelin application to Reelin KO mouse brain slices can promote dendritic growth (Nichols and Olson, 2010). The role of alterations in neurite growth and branching and the associated ASD-linked genes, are described in detail later.

## Alterations in Synaptic Components in ASD

Synapses are highly specialized structures required for signal transduction and plasticity within neuronal networks, making up the functional contact sites between neurons. A synapse is composed of the axon terminal, the presynapse, a synaptic cleft which contains adhesion proteins and the extracellular matrix, and the postsynaptic density with receptors on a target neuron’s dendrites. The presynapse contains mitochondria and is characterized by a pool of synaptic vesicles filled with neurotransmitters. Action potentials arriving at the axon terminal mediate calcium influx, which causes the vesicles to fuse with specialized regions of the plasma membrane called active zones to release their content into the synaptic cleft. A precise coupling between the electrical stimulus (action potential) and release of neurotransmitter is crucial for proper signal transmission to the postsynaptic neuron. The postsynapse is characterized by the presence of receptors that bind neurotransmitters released from the presynapse, which initiates signaling cascades that ultimately propagate the electrical signal in postsynaptic neuron. Receptors are clustered in a region called the postsynaptic density (PSD) in the excitatory synapse.

Synapses are classified as excitatory and inhibitory, depending on whether they use glutamate or γ-aminobutyric acid (GABA) as their main neurotransmitter, respectively. Additionally, the formation and structure of excitatory and inhibitory synapses is uniquely different. Whereas excitatory synapses made on dendritic spines, inhibitory synapses are formed directly on the dendritic shaft. Additionally, both excitatory and inhibitory synapses have distinct proteomic profiles to specialize each synapse with the appropriate receptors and signaling molecules. Synaptic dysfunctions, whether they arise from functional mutations in pre- or postsynaptic proteins, are a common underlying pathology in ASD and are discussed in detail below.

### Presynaptic Proteins Linked to ASD

Neurexins (*NRXN*) are synaptic adhesion proteins that localize to the membrane of the presynapse and bind Neuroligins (NLGNs), which are localized on the postsynaptic membrane. There are three genes within the NRXN family (*NRXN1*, *NRXN2*, and *NRXN3*) with multiple mutations or copy number variations having been identified in NRXN family members in ASD diagnoses (Feng et al., 2006; Autism Genome Project Consortium et al., 2007; Bremer et al., 2011; Yangngam et al., 2014). *NRXN1* mutations have also been identified in multiple neuropsychiatric disorders, including Tourette syndrome, schizophrenia, ADHD and bipolar disorder (Stone et al., 2008; Guilmatre et al., 2009; Zhang et al., 2009; Sundaram et al., 2010; Lionel et al., 2011).

*NRXN1α* KO mice exhibit reduced excitatory synaptic strength, with a decrease in the input–output relationship of evoked postsynaptic potentials and miniature excitatory postsynaptic current (mEPSC) frequency (Etherton et al., 2009). In behavioral tests, *NRXN1* KO mice display an increase in grooming behavior, however no change in spatial learning or social behavior (Etherton et al., 2009). In *α-NRXN* triple KO mice, where NRXN1α/2α/3α were deleted, synapse formation was normal but α-Nrxns were required to couple Ca^2+^ channels to vesicle exocytosis (Missler et al., 2003).

The synapsins (*SYN*) are a family of presynaptic proteins involved in vesicle-mediated neurotransmitter release and neurite outgrowth (Rosahl et al., 1995). In mammals, the synapsin family of proteins contains three members (synapsin 1, synapsin 2, and synapsin 3). Primary neuron cultures from *SYN1/2/3* triple KO mice display severely altered synaptic vesicle localization and a significant decrease in the number of synaptic vesicles (Fornasiero et al., 2012). Additionally, mutations in *SYN1* (A51G, A550T, Q555X and T567A) have been identified in a family with epilepsy and autistic phenotypes, suggesting its link as a causative factor for ASD (Fassio et al., 2011). Additionally, the nonsense mutation, Q555X, reduces CaMKII and MAPK/ERK activity, which regulates synaptic vesicle trafficking and neurite growth, and the A550T and T567A missense mutations have been shown to impair synapsin localization to the presynapse (Fassio et al., 2011).

In synapsin KO mice, studies have shown behavioral deficits in social novelty and avoidance behavior in social approach as well as epileptic activity, all of which are typical in ASD (Greco et al., 2013; Ketzef and Gitler, 2014). *SYN1/3* double KO mice show an impairment in social transmission of food preference, while *SYN1/2* double KO mice have a decrease in environmental interest. Additionally, *SYN2* KO mice have impairments in social recognition tests and display an increase in repetitive self grooming (Greco et al., 2013). These findings suggest that synapsins play an important role in the underlying pathology leading to behavioral phenotypes typical in ASD.

### Postsynaptic Proteins Linked to ASD

Neuroligins (*NLGN*) are cell adhesion proteins that localize to the postsynaptic membrane and play an integral function in synapse formation via binding their presynaptic partners, *NRXN*s. In humans NLGN family is composed of *five* genes (*NLGN1/2/3/4/4Y*). The large extracellular domain of Nlgns have a high sequence similarity to acetylcholinesterase and is required to bind to β-Nrxn during synapse formation (Dean and Dresbach, 2006). Nlgns-1, -3 and -4 are localized to excitatory synapses, while Nlgn2 is found at inhibitory synapses (Missler et al., 2003) Previous studies have shown that overexpression of Nlgn1 can increase excitatory synaptic strength and the synaptic NMDAR/AMPAR ratio both *in vitro* and *in vivo* (Schnell et al., 2012). Conversely, Nlgn2 overexpression increases inhibitory synaptic strength. Additionally, Nlgn1 expression can promote synapse maturity but does not induce synapse formation of glutamatergic synapses (Chubykin et al., 2007; Schnell et al., 2012).

The Shank family are scaffolding proteins localized to the postsynapse and interact with NMDA receptors as well as Nlgn-Nrxn complexes. The Shank protein can interact with multiple important postsynaptic proteins including the actin cytoskeleton via ankyrin repeats, Ca^2+^ signaling via calpain/calmodulin and the glutamate receptor-interacting protein (GRIP) through its SH3 domain (Lim et al., 1999; Yoo et al., 2014). *SHANK1*, *SHANK2* and *SHANK3* comprise the Shank family of proteins and they are found throughout the brain, however each isoform varies in its distribution.

*SHANK3* was the first gene in the Shank family of synaptic scaffolding proteins to be linked to ASD. During spine formation, Shank3 is an important scaffolding protein and loss of Shank3 *in vitro* results in a decrease in both the length and density of spines. Conversely, over-expression of Shank3 results in more mature and larger spines (Betancur et al., 2009). *SHANK3* resides on chromosome 22q13.3, and ASD-linked region with deletions linked to Phelan-McDermid syndrome. This syndrome is characterized by developmental delay, severely impaired language, ASD and ID (Phelan, 2008).

Transgenic mice harboring various *SHANK* mutations or deletions have elucidated Shank’s in synapse formation, function and its role in ASD. Transgenic mice with genetic KO of the longest isoform of *SHANK3*, have been shown to recapitulate the phenotypes observed from *SHANK3* mutations in humans. Although social impairments in these mice have varied, they have all shown increased repetitive grooming, a behavior typical in ASD mouse models (Bozdagi et al., 2010; Peça et al., 2011; Wang et al., 2011; Yang et al., 2012).

Postsynaptic density protein-95 (PSD-95, DLG4) is an important postsynaptic scaffolding protein that localizes to excitatory synapses. PSD-95 is composed of three PDZ domains that target it to the postsynapse, an SH3 domain, and a guanylate kinase domain on its C-terminal. Nlgn, NMDARs and potassium channels all interact with the PDZ domains on PSD-95. PSD-95 localizes to spine heads in excitatory synapses and has been shown to promote synapse stabilization. The E3 ubiquitin ligase, murine double minute-2 (Mdm2) ubiquitinates PSD-95 and subsequently binds with protocadherin-10 (Pcdh10), sending it to the proteasome to be degraded. Pcdh10 is an ASD-linked gene (Morrow et al., 2008) and its expression is regulated via the interaction of myocyte enhancer factor-2 (MEF2) and FMRP (Tsai N. P. et al., 2012). Additionally, in FMRP KO neurons, dysregulated Mdm2 prevents MEF2-induced PSD-95 ubiquitination and synapse elimination (Tsai N. P. et al., 2012), providing evidence for altered activity-dependent synapse elimination in an ASD model. PSD-95 KO mice (*DLG4* KO) show multiple behavioral and molecular abnormalities that are linked to ASD pathology. *DLG4* KO mice display impaired communication and social interactions, decreased motor coordination, as well as increased anxiety and repetitive behaviors. Additionally, *DLG4* KO mice display defects in dendritic spine morphology in the amygdala and aberrant expression of multiple synapse-related genes in the forebrain (Feyder et al., 2010).

Gephyrin is a key postsynaptic scaffolding protein in inhibitory synapses. Gephyrin’s three major domains include a N-terminal G-domain, a C-terminal E-domain, with a large linker region connecting the two domains. Gephyrin interacts with glycine receptors with high affinity and the α and β subunits of the GABA_A_ receptor. Nlgn2 binds to gephyrin via a cytoplasmic motif region and activates collybistin, and the Nlgn2/gephyrin/collybistin complex is necessary for clustering of inhibitory receptors (Poulopoulos et al., 2009). In gephyrin KO mice, there is a decrease in GABA_A_ and glycine receptor clustering, whereas glutamate receptor localization remains normal (Kneussel et al., 1999; Grosskreutz et al., 2003).

Rare exonic deletions within the gephyrin (*GPHN*) gene have been reported in subsets of patients with ASD (Lionel et al., 2013). A *de novo* 273 kb deletion in *GPHN* displayed developmental delay, cyclical seizures, repetitive behaviors increased anxiety and obsessive compulsive disorders, in one family (Lionel et al., 2013).

### Adhesion Complexes Involved in ASD

CNTNAP2 is a member of the Nrxn superfamily and plays an important role regulating the clustering of potassium channels and neuron-glia interactions. CNTNAP2 was first linked to ASD Amish children presenting with developmental disorders, seizures, and impaired language were found to have a homozygous mutation in CNTNAP2 (Strauss et al., 2006). After genetic deletion of CNTNAP2, mice show significantly decreased numbers of dendritic spines as well as a decrease in the GluA1 subunit of AMPARs. Additionally, an aggregation of GluA1 was observed in the cytoplasm, suggesting that synaptic deficits may be in part due to a trafficking problem (Varea et al., 2015). CNTNAP2 KO mice show common ASD behavioral phenotypes, including repetitive movements as well as social deficits (Peñagarikano et al., 2011).

**Figure 3:**
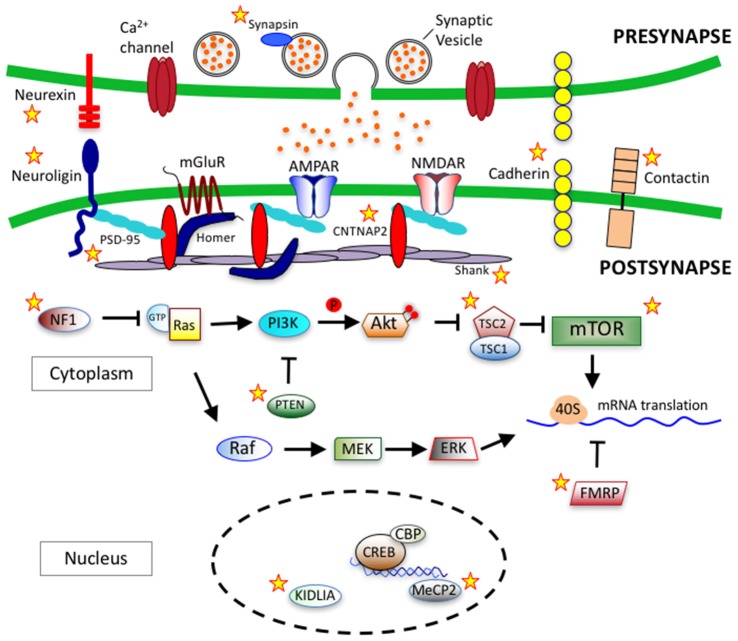
Synaptic proteins and signaling pathways linked to autism spectrum disorder (ASD). ASD-linked synaptic proteins and signaling pathways that relate to synaptogeneis and synaptic function. Stars mark ASD-linked proteins discussed in this review article. *Abbreviations*: AMPAR, AMPA receptor; NMDAR, NMDA receptor; mGluR, metabotropic glutamate receptor; PSD-95, postsynaptic scaffolding protein 95 kDa; CNTNAP2, contactin-associated protein-like 2 gene; PI3K, phosphoinositide-3 kinase; Ras, RhoGTPase; GTP, Guanosine-5′-triphosphate; NF1, Neurofibromatosis type 1; PTEN, phosphatase and tensin homolog; Akt, serine/threonine specific kinase; TSC, tuberous sclerosis complex; mTOR, mammalian target of rapamycin; Raf, Rapidly accelerated fibrosarcoma serine threnonine kinase, MEK, Mitogen-activated protein kinase kinase; ERK, extracellular signal–regulated kinase; 40S, ribosomal subunit; FMRP, fragile-X mental retardation protein; CREB, cAMP response element-binding protein; CBP, CREB binding protein; MeCP2, methyl CpG binding protein 2; KIDLIA, *KIAA2022* gene with intellectual disability (ID) and language impairment in autism; P, phosphate group.

Cadherins (*CDH*) are transmembrane proteins that function in cell-cell adhesion and play roles in neuron migration, dendritic growth, spine morphology, synaptogenesis and synapse remodeling (Tan et al., 2010; Redies et al., 2012; Bian et al., 2015; Egusa et al., 2016; Gilbert et al., 2016). In genome-wide association studies, common variants in *CDH9* and *CDH10* genes on chromosome 5p14.1 have been identified to link with ASD (Wang et al., 2009). Additionally, deletions within 16q23 in *CDH13* have also been identified in ASD patients (Sanders et al., 2011). Neural cadherin (N-cadherin) is a calcium dependent cell-cell adhesion glycoprotein that plays important roles in neurodevelopment (Garcia-Castro et al., 2000). δ-catenin, a neuron specific member of the p120 family of catenins, is a known interactor of the cytoplasmic juxtamembrane region of N-cadherin (Lu et al., 1999). The δ-catenin gene, *CTNND2*, is candidate gene in ASD and functions within a protein network that has a major role dendritic and spine growth and dynamics (Bian et al., 2015; Turner et al., 2015; Yuan et al., 2015). Over-expression of δ-catenin induces dendritic and spine growth in primary neuron cultures (Martinez et al., 2003; Arikkath et al., 2009), while knockdown of δ-catenin serves to impair dendritic growth (Elia et al., 2006; Arikkath et al., 2008). Figure 3 depicts key synaptic proteins and signaling pathways linked to ASD.

## Dysregulation of Synaptic Plasticity and Neuronal Activity in ASD

Based on the different ASD-linked pathways discussed previously, it can be inferred that changes in synaptic plasticity via altered synaptic strength and/or number, may be an underlying pathology in ASD patients and mouse models. Interestingly, many ASD-linked mutation results in altered gene transcription and protein synthesis of synaptic related transcripts, effects which can also be observed with changes in neuronal activity (Kelleher and Bear, 2008; Akins et al., 2009; Darnell et al., 2011; Qiu et al., 2012; Gilbert and Man, 2014).

Initial findings from FMR1 mutant mice, the mouse model of FXS, did not show impairments in LTP using a standard HFS paradigm (Godfraind et al., 1996; Li et al., 2002). However, using a low threshold stimulation protocol, LTP induction was reduced in multiple brain areas, including the hippocampus and somatosensory cortex (Larson et al., 2005; Zhao et al., 2005; Lauterborn et al., 2007). Additionally, mGluR-LTP was impaired in the basolateral amygdala and visual cortex (Wilson and Cox, 2007; Suvrathan et al., 2010). These findings provide evidence that different brain regions or synapse-specific deficits occur in mice with loss of FMRP. The most prominent change in synaptic plasticity has been observed with enhanced mGluR-LTD the cerebellum and in CA1 of the hippocampus (Huber et al., 2002; Koekkoek et al., 2005). An enhancement of LTD in the CA1 region has led to the theory that synaptic loss of FMRP produces increased signaling through mGluRs (Huber et al., 2002; Bear et al., 2004; Osterweil et al., 2010). Additionally, genetic reduction, or mGluR5 inhibition, rescues behavioral deficits in *FMR1* mice (Dolen et al., 2007; Michalon et al., 2012). These rescue experiments therefore suggest the ability to reverse some of the impairments in FXS patients. The different impairments in synaptic function found across various brain regions in *FMR1* mice provide evidence of a correlation between impaired synaptic plasticity and behavioral deficits in ASD. These studies also provide evidence that the *FMR1* KO mouse may be a good model to explore the pathophysiology of ASD and investigate possible treatment strategies (Bhakar et al., 2012).

Synaptic plasticity has been studied in great detail in the *UBE3A* maternally deficient mice, the Angelman syndrome mouse model. In the CA1, HFS-LTP is reduced in transgenic mice and the reduction in CA1 LTP could be rescued with stronger stimulation protocols (Weeber et al., 2003). These findings indicate that Ube3a’s role in synaptic plasticity may be as a modulator of LTP, not necessarily required for LTP induction. In the same study, a reduction in calmodulin-dependent protein kinase II (CaMKII) activity was observed in transgenic mice, and genetic reduction of CaMKII’s inhibitory autophosphorylation rescued deficits in LTP and learning and memory tasks in transgenic animals (van Woerden et al., 2007). These findings provide evidence that altered CaMKII activity mediates the impairment in synaptic plasticity in *UBE3A* deficient mice; however, it remains to be elucidated how loss of UBE3A alters the activity of CaMKII.

Both of the *TSC1* or *TSC2* KO mice are embryonic lethal; however, mice harboring *TSC1* or *TSC2* heterozygous mutations display synaptic dysfunction and cognitive impairments (Kobayashi et al., 2001; von der Brelie et al., 2006; Ehninger et al., 2008). In *TSC2* heterozygous rats, LFS-LTD was decreased and L-LTP was enhanced in the CA1 of the hippocampus; however, E-LTP was unaffected (von der Brelie et al., 2006; Ehninger et al., 2008), suggesting changes in protein synthesis pathways necessary for L-LTP expression. Additionally, in *TSC2* heterozygous mice, mGluR-LTD was decreased but LFS-LTD was unchanged (Auerbach et al., 2011). A reduction in mGluR-LTD in *TSC2* heterozygous animals opposes what is observed in the *FMR1* KO mice; however, mGluR-LTD in both animals don’t show sensitivity to protein synthesis inhibitors (Auerbach et al., 2011). An additional study using mice with a specific deletion of *TSC1* in cerebellar Purkinje cells displayed deficits in social interactions, an increase repetitive behavior as wells as defects in ultrasonic vocalizations. These animals however were not investigated for changes in synaptic plasticity in the cerebellum (Tsai P. T. et al., 2012). These findings suggest that impaired synaptic plasticity is a major pathology in TSC mouse models of autism, underlying the observed deficits in social interaction in both in *TSC1* and *TSC2* heterozygous animals.

In *MECP2* KO mice, reductions in LTP and LFS-LTD in CA1 of the hippocampus have been reported. It’s interesting to note that younger mice (~3–5 weeks of age) show no impairments in synaptic plasticity, indicating that impaired synaptic plasticity correlates with the delayed deficits observed in human RTT patients (Chen et al., 2001; Asaka et al., 2006). A mouse model containing a truncation of MeCP2 (MeCP2308/Y) showed a reduction in LTP as well as paired-pulse stimulation, but no changes in mGLuR-LTD (Shahbazian et al., 2002; Weng et al., 2011). Interestingly, another study has shown that impaired LTP in CA1 of the hippocampus could be rescued by genetically reintroducing MeCP2 (Guy et al., 2007; Weng et al., 2011).

Shank proteins regulate levels and modulate the signaling of both metabotropic and ionotropic glutamate receptors at the synapse, and synaptic plasticity has been studied in multiple Shank3 mouse models (Tu et al., 1999; Uchino et al., 2006). Investigation of mEPSCs, paired pulse ratio (PPF and PPD), input/output curves and population spikes have indicated different abnormalities in synaptic transmission in hippocampal CA1 synapses of mice with different Shank3 mutations (Wang et al., 2011). Additionally, hippocampal LTP is reduced in the CA1 of the hippocampus in subsets of mice with Shank3 truncations, as well as LFS- LTD and mGLuR-LTD (Bozdagi et al., 2010; Bangash et al., 2011; Wang et al., 2011).

It has been hypothesized that disruptions in neuronal circuits involved with language and social behavior in subtypes of autism may be caused by unbalanced high levels of excitation, or disproportionately weak inhibition (Rubenstein and Merzenich, 2003; Gao and Penzes, 2015). With a more excitable cortex, the brain would have wide-ranging abnormalities in perception, memory, cognition and motor control, and would be highly susceptible to epilepsy (Rubenstein and Merzenich, 2003). In support of this theory, a decrease in glutamate decarboxylase 67 (*GAD67*) mRNA in autistic cerebellar Purkinje cells has been identified in human autism samples (Yip et al., 2007). Additionally, another study has shown a decrease in GABAergic inhibition in autism patient samples (Hussman, 2001), indicating that decreased GABAergic inhibition may disrupt the excitation/inhibition balance within neuronal networks in autism.

As a novel form of synaptic regulation opposing the Hebbian type plasticity, homeostatic synaptic plasticity (HSP) is a negative feedback response that serves to compensate for changes in network activity (Turrigiano et al., 1998; Hou et al., 2008, 2011, 2015; Yu and Goda, 2009; Pozo and Goda, 2010; Wang et al., 2012; Gilbert et al., 2016). In response to global decreases or increases in network activity from a homeostatic set-point, synaptic strengths are scaled up or down, respectively (Turrigiano et al., 1998). HSP may be important for maintaining network activity homeostasis and avoiding potential epileptogenic states and sleep may be necessary for synaptic homeostasis (Tononi and Cirelli, 2003; Kuhn et al., 2016). The studies in *Drosophila* for example, have shown that the size and number of synapses decrease after sleep and increase within a few hours of waking (Gilestro et al., 2009). Interestingly, Nlgns regulate levels of glutamatergic and GABAergic currents after sleep deprivation, indicating they play an important role in this sleep-dependent HSP (Huber et al., 2004; Gilestro et al., 2009; El Helou et al., 2013) and Nlgn defects could result in disturbances in sleep and circadian rhythms, a common disorder found in ASD patients (Bourgeron, 2007). FMRP, the RNA-binding protein involved with regulating dendritic protein translation, has also been shown to be required for increases in synaptic strength after neuronal activity blockade or application of retinoic acid in the mouse hippocampus, indicating that some symptoms of FXS may be due to impaired HSP (Soden and Chen, 2010). Release of brain-derived neurotrophic factor (BDNF) from postsynaptic neurons has previously been indentified to be required for a retrograde homeostatic up-regulation of presynaptic function. Increased BDNF levels in ASD patient blood samples has been observed as well as higher plasma levels of serotonin and N-acetylserotonin (NAS), a potent agonist of the BDNF receptor tyrosine receptor kinase B (TrkB; Jang et al., 2010; Halepoto et al., 2014; Kasarpalkar et al., 2014; Pagan et al., 2014). Excess levels of NAS could therefore increase TrkB-induced PI3K signaling, resulting in increased protein translation, similar to findings observed with mutations in components of the mTOR pathway.

## Final Conclusions

ASD is diagnosed at the behavioral level with the presentation of its core phenotypes of impaired social interactions, restrictive interests and repetitive behaviors. Although the etiological factors of ASD are highly heterogeneous, recent research has strongly pointed to common cellular events that are impaired in ASD, including neurogenesis, morphogenesis, synapse maturation and synaptic plasticity. In this regard, loss-, or gain-, of-function mutations in single genes that are causative for ASD have given researchers unique opportunities to make important mechanistic insights. A common theme emerging within the field is that in the developing brain, alterations in dendritic growth, synapse formation and synaptic function result in neuronal network dysfunction, ultimately leading to complex social and cognitive dysfunction. Further elucidation into these pathways, as well as advancements in gene therapies and targeted drugs to modulate these processes, could provide exciting and promising new therapies for the treatment of ASDs.

## Author Contributions

JG and H-YM wrote and edited the manuscript.

## Conflict of Interest Statement

The authors declare that the research was conducted in the absence of any commercial or financial relationships that could be construed as a potential conflict of interest.
